# Associations between anthropometric parameters and immune-phenotypical characteristics of circulating Tregs and serum cytokines

**DOI:** 10.1007/s00011-023-01777-1

**Published:** 2023-09-02

**Authors:** Timo Schmitz, D. Freuer, C. Meisinger, J. Linseisen

**Affiliations:** Epidemiology, Medical Faculty, University Hospital Augsburg, University of Augsburg, Stenglinstraße 2, 86156 Augsburg, Germany

**Keywords:** BMI, Body fat, Visceral fat, Cytokines, Flow cytometry, Regulatory T cells

## Abstract

**Objective:**

To investigate the associations between several anthropometric parameters and regulatory T cells (Tregs) and circulating cytokines in a population-based cohort.

**Methods:**

Between 2018 and 2021, a total of 238 participants were examined up to three times within the scope of the MEGA study in Augsburg, Germany. Tregs were analyzed using flow cytometry and the serum concentrations of 52 cytokines were determined. Anthropometric parameters were measured, using also bioelectrical impedance analysis: body mass index (BMI), relative total body fat, relative visceral adipose tissue (rVAT), waist circumference (WC), waist-to-hip ratio (WHR) and body fat distribution. Associations were analyzed using linear mixed models with random intercept (Tregs) and conventional linear regression models (cytokines).

**Results:**

WC and WHR were inversely associated with the general Treg subset. Four parameters (BMI, rVAT, WC, and WHR) were inversely associated with the conventional Treg population. Three cytokines showed a particularly strong association with several anthropometric parameters: the cutaneous T-cell attracting chemokine was inversely associated with anthropometric parameters, while hepatocyte growth factor and interleukine-18 showed positive associations.

**Conclusions:**

Anthropometric measures are associated with Tregs and serum cytokine concentrations revealing new important interconnections between obesity and the adaptive immune system.

**Supplementary Information:**

The online version contains supplementary material available at 10.1007/s00011-023-01777-1.

## Introduction

Within the last decades, overweight and obesity became one of the major health risks in western societies and worldwide [1, 2]. Many diseases are supposed to be causally associated with increased body mass index (BMI) and total body fat (TBF) in general, and visceral adipose tissue (VAT) in particular [3–6]. Since recently, it became more obvious, that the impact of obesity on the immune system explains a major part of the association between obesity and chronic disease [7]. At first, influences especially on the innate immune system (e.g., macrophages) were described in various studies, but more recent studies also reported connections with the adaptive immune system [7]. Still, the overall interplay between obesity and changes in the functionality of the immune system are far from being understood completely. To gain a deeper understanding of this relationship the present study investigates the associations between various anthropometric parameters and regulatory T cells (Tregs) in a population-based cohort with repeated measurements. In addition, serum cytokines were examined to inform the results for Tregs with data at the protein level.

## Methods

### Study population and data collection

This analysis is based on data from the MEGA study, a single center, population-based cohort study conducted between 2018 and 2021 in Augsburg, Germany. Participants between 25 and 65 years were recruited and examined up to 4 times within a time period of 9 months (baseline visit, follow-up visit after 1 and 6 months, final visit after 9 months). For this study, only patients without fever (body temperature ≤ 38.5 °C in the last 24 h) and without antibiotics or immunosuppressant use in the last 3 months were included. All examinations were performed in a 12 h overnight fasting state. All study participants gave written informed consent. Methods of data and biospecimen collection have been approved by the ethics committee of the Ludwig-Maximilians-Universität München, and the study was performed in accordance with the Declaration of Helsinki. The study was registered at the German Register of Clinical Studies (DRKS) with the project number DRKS00015784.

### Data collection

Next to a variety of different examinations, every visit included the collection of venous blood samples and an extensive survey addressing comorbidities and existing diseases, risk factors, and mental health. Among other topics, the following information was obtained during the face-to-face interview: total daily alcohol consumption, smoking status (current smoker, never smoker, previous smoker), and education (no professional education, professional education, academic education).

At each study visit, anthropometric measurements were made (height, weight, waist circumference, hip circumference) and a body composition analysis via bioelectrical impedance analysis (BIA, SECA mBCA515 device) was conducted. For the present analysis, the following parameters were used as exposure variables: body mass index (BMI, kg/m^2^), relative total body fat (rTBF, in % of body weight), relative visceral adipose tissue (rVAT, in % of body weight), waist circumference (WC, in cm), waist-to-hip ratio (WHR) and body fat distribution (BFD; visceral body fat mass divided by total body fat mass).

All examination steps were carried out by trained study nurses in accordance with previously defined standard operating procedures.

### Flow cytometry

**Fig. 1:**
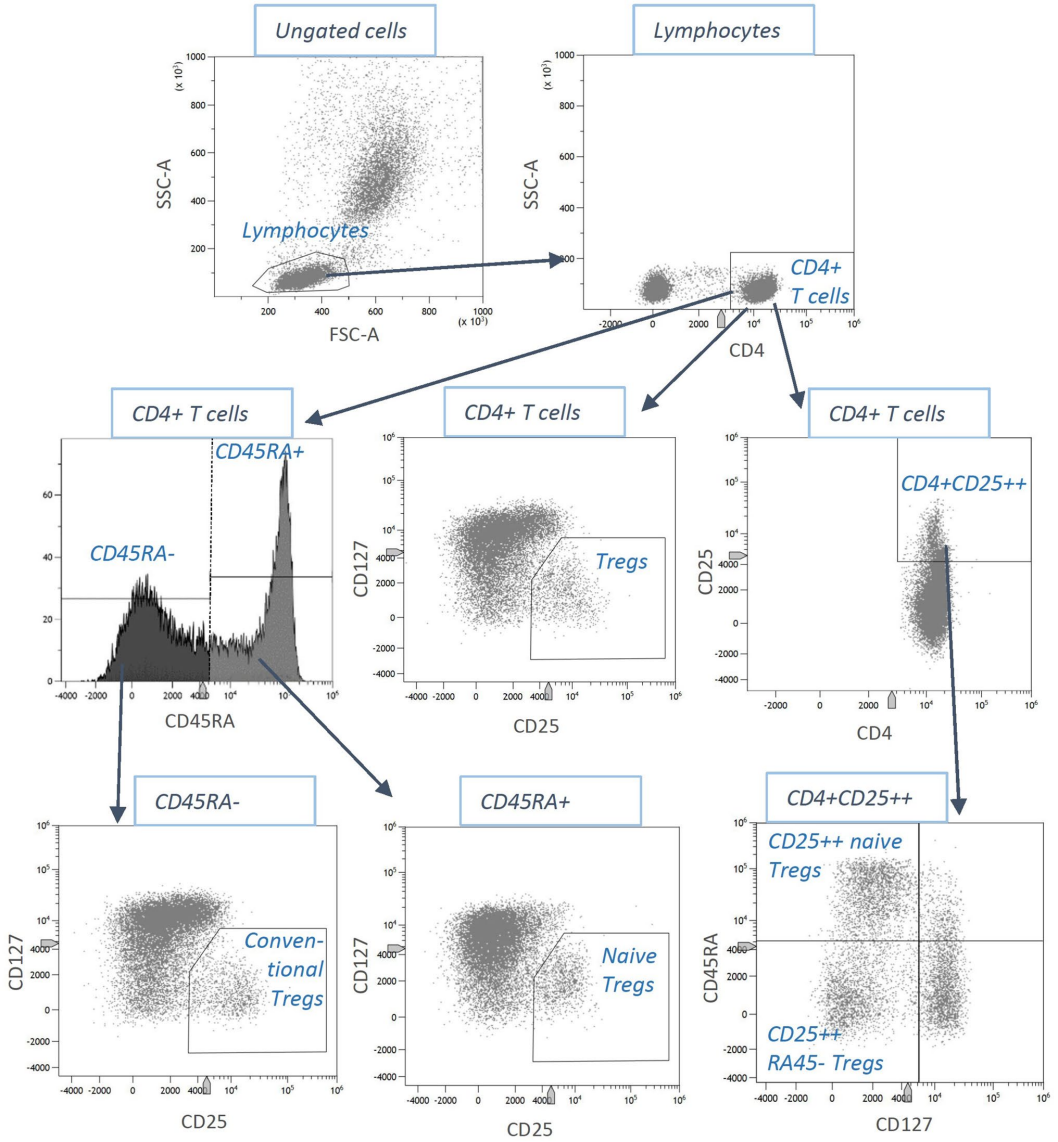
Gating strategy for the Treg panel. In the first step, T helper cells were identified by their expression of CD4. Then, different CD4 + T cell subsets were analyzed using the following antibodies: anti-CD25, anti-CD127, anti-CD45RA. The identified cell subsets were quantified by calculating their proportion of the parent gate cells

Venous EDTA blood samples were used for the flow cytometry measurements (Cytoflex LX flow cytometer, 6 lasers, Beckman Coulter GmbH, Krefeld, Germany). In a first step, erythrocytes were lysed by using VersaLyse Lysing Solution (Beckman Coulter) and subsequently immune cells were isolated in several washing steps. Leucocytes were treated with an FC receptor block, which avoids non-specific antibody binding. Then, antibody staining was performed with fluorescence-labelled liquid antibodies. The following antibodies were used for the analysis of Tregs: anti-CD4, anti-CD25, anti-CD127 and anti-CD45RA. The best concentration of antibodies was predetermined by titration. The antibody-coupled immune cells were fixed using IO-Test 3 Fixative Solution (Beckman Coulter). In a final step, the T cells were analyzed via flow cytometry. The gating was performed using Kaluza software (Beckman Coulter). Figure 1 displays the applied gating strategy. For each T cell subpopulation, the relative frequency of the respective cells (as a share of the total cell count of the parent gate) was estimated and used for the statistical analysis.

### Cytokines measurement

Concentrations of 52 cytokines were measured in serum using the Bio-Plex Pro™ Human Cytokine Screening Panel, ICAM-1 set and VCAM-1 set, plus the Pro™ Human TH17 cytokine sCD40L set and IL31 set (all Bio-Rad Laboratories Inc., Hercules, California, USA). The assays were carried out according to the manufacturer’s specifications. In brief, 50 µl of diluted magnetic beads were placed in the wells and washed 2 times. Then the standard samples, blank and controls were pipetted into the respective well and incubated between 30 and 60 min on the shaker at 850 ± rpm at room temperature. This was followed by a threefold wash step, the addition of the diluted detection AB and another 30 min incubation (all incubation steps were carried out at room temperature). Subsequently and after another wash step, streptavidin PE was added and incubated for 10 min. Afterwards, the plate was washed 3 more times and re-suspended with 125 µl. For the measurements, Luminex xMAP technology instrument and Bioplex Manager Software was used. The measurements were performed in six batches. The technically related variations between the plates were corrected using intensity normalization (defined as x_norm = x − *x̃*_plate + *x̃*_overall, where *x̃*_plate and *x̃*_overall represent the plate-specific and overall medians).

For many measures the cytokine concentrations were below the limit of detection. For this analysis, we excluded all cytokines with missing values (in general due to concentrations below the limit of detection) in 25% or more of all measures. Consequently, linear regression models were calculated for 25 cytokines. For these cytokines, missing values due to concentrations below the limit of detection were set to the lower detection limit. 27 cytokines have not been used for the statistical analysis. In the supplementary material (Table S2) we give an overview of all cytokines measured and the number of missing values.

### Sample size

A total of 238 participants were examined at least once. The flow cytometry measures started with a short delay (after the start of the study), so baseline data for Tregs were available in 215 patients. For both follow-up visits (after 6-months and after 9-months), there was flow cytometry data available for 200 participants each. So overall, 615 measures could be included into the mixed model analyses.

**Fig. 2:**
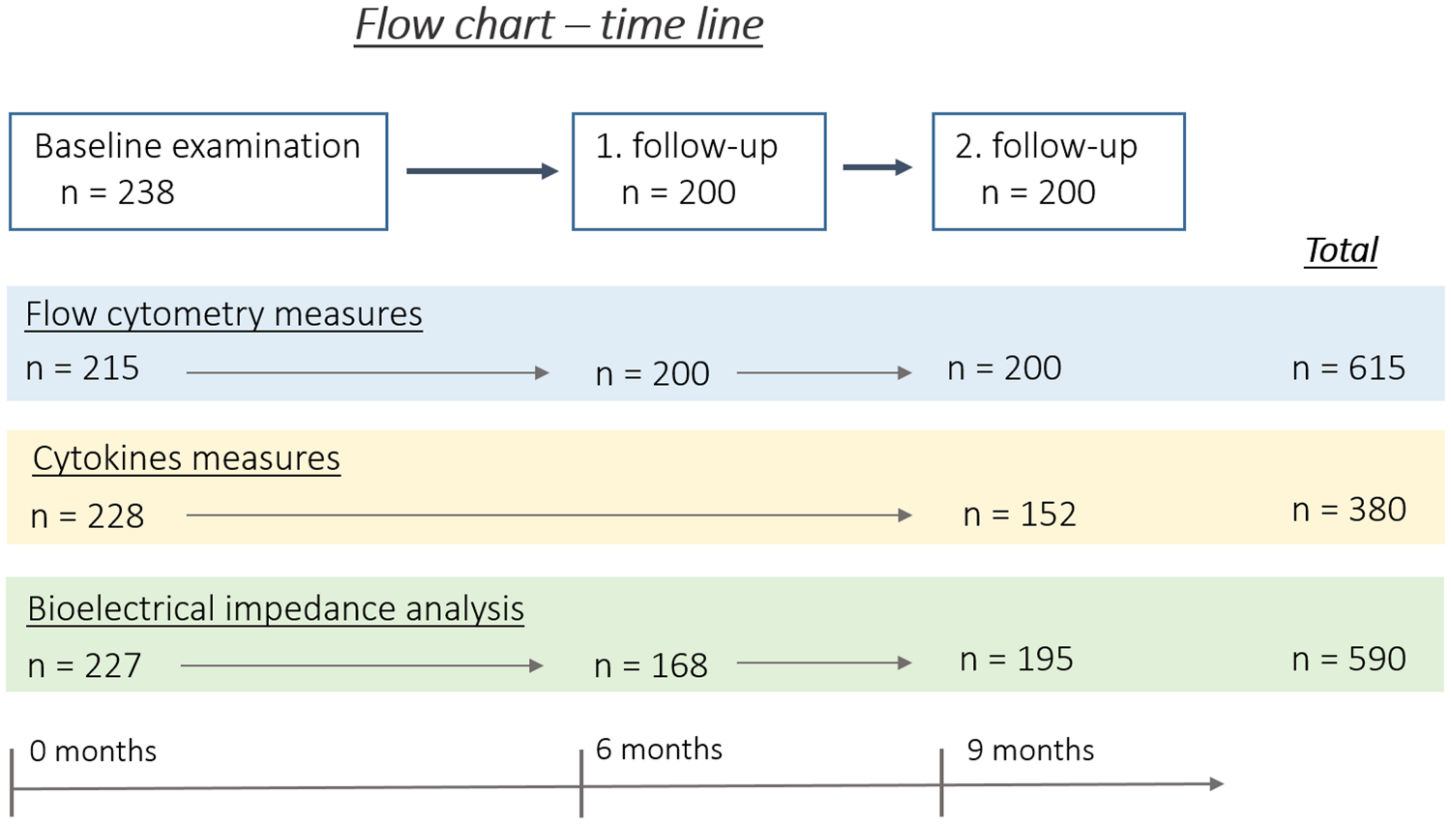
Flow chart and time line of the included participants and measures for the analysis of Tregs (flow cytometry) and cytokines

Determination of cytokine concentrations was performed for baseline visits (n = 228) and 9-months follow up (n = 152) only. Consequently, a total of 380 measures was considered for the linear regression models. Due to restriction caused by the Covid-19 pandemics, the bioelectrical impedance analysis had to be temporarily removed from the examination. Consequently, 590 measurements of rTBF, rVAT and body fat distribution were available (baseline: 227, follow-up visits after 6-months 168, follow-up visits after 9-months: 195). Information on BMI, waist circumference and waist-to-hip ratio was available for every participant and for every examination visit. An overview of the sample sizes and the number of included measures is displayed in Fig. 2.

### Statistical analysis

In general, categorical variables were compared using Chi-square tests and the results were presented as absolute frequencies with percentages. For normally-distributed continuous variables, Student’s t-tests were used. For continuous variables that were not normally-distributed we used nonparametric tests. The results were presented as mean and standard deviation (SD) or median and inter-quartiles range (IQR).

### Mixed models with random intercept

For the majority of participants there was data on Treg subpopulations and body composition (BMI, body fat, visceral body fat) for three different time points available. An appropriate statistical method for this longitudinal structure of the data are random intercept models with identity link based on Maximum Likelihood estimation. We calculated such models to examine the associations between Treg subpopulations (outcomes) and parameters of body composition (exposures). For the Treg subpopulations, we used the relative proportion of these cells on the corresponding parent gate. We standardized the outcome as wells as the exposure variables to ensure comparability of the obtained effect sizes. In order to avoid excessive impact of single outliers, we removed observations with a deviation from the mean of greater than three standard deviations. According to literature review, all models were adjusted for the following potential confounder variables: sex, age, education, smoking status, alcohol consumption and hypothyroidism. Normal distribution of the regression residuals was checked graphically and considered to be fulfilled sufficiently.

### Linear regression models

Data on cytokine concentrations was available for 2 time points (baseline, 9-months-follow-up). For that reason conventional linear regression models instead of mixed models were calculated. Only cytokines with missing values of less than 25% were analyzed. The models were adjusted for the same confounding variables as the mixed models for Tregs and additionally for the corresponding visit (baseline, follow-up). Just like for the mixed models, the exposure and outcome variables were standardized and outliers exceeding a deviation of more than 3 SD were removed from the analysis. Again, normal distribution of the regression residuals was checked graphically.

**Fig. 3:**
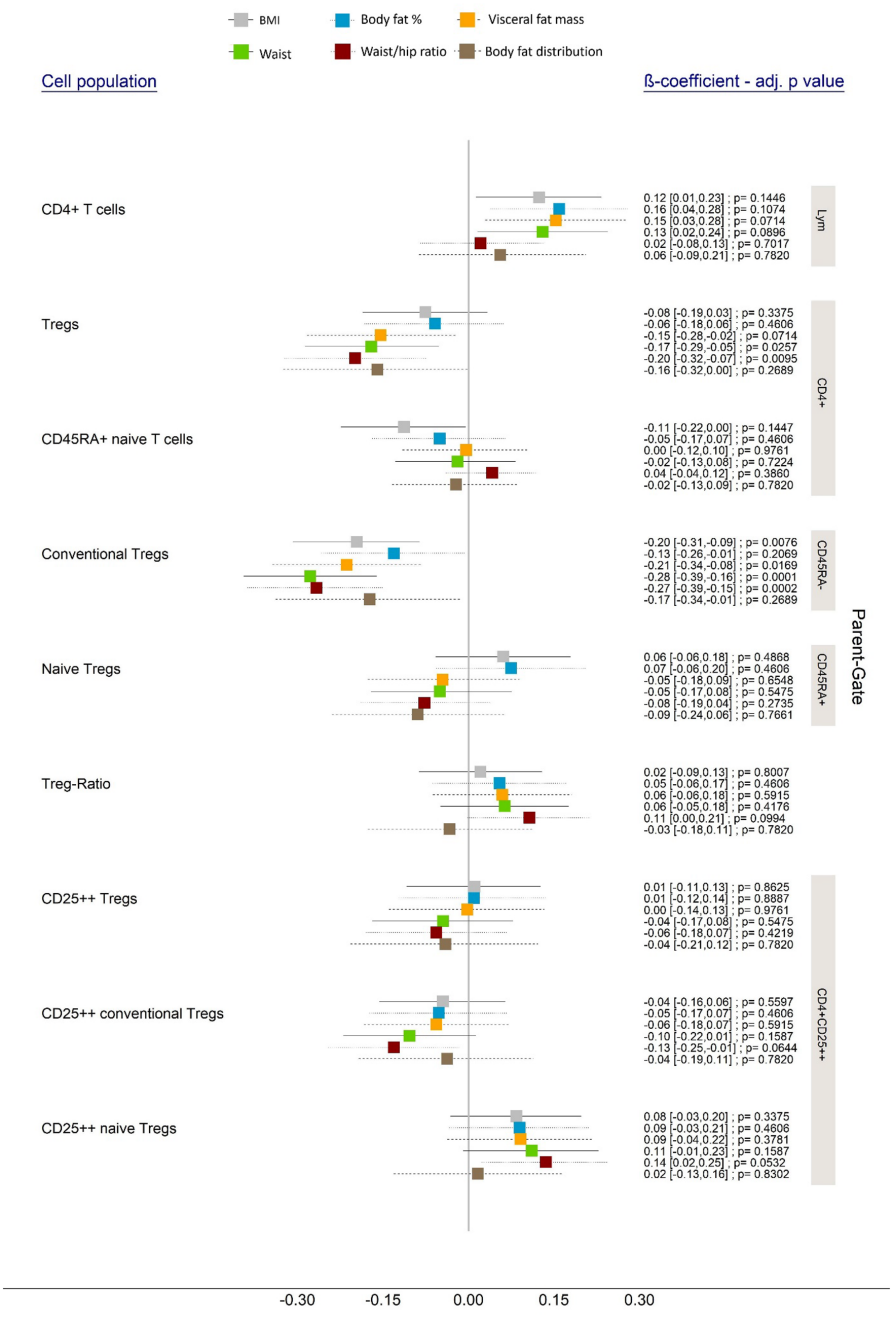
Associations between Treg subsets and anthropometric parameters. The figure displays the results of linear mixed models with random intercept and adjusted for sex, age, education, smoking status, alcohol consumption and hypothyroidism. Both, the exposure variables and the outcome were standardized. The results are presented on the right side (estimated β-coefficients with 95% CI, and FDR adjusted p values) and are graphically illustrated by the forest plot. The specific cell populations are shown on the left side and the respective parent gate is represented by the gray rectangles on the right edge of the figure

**Fig. 4:**
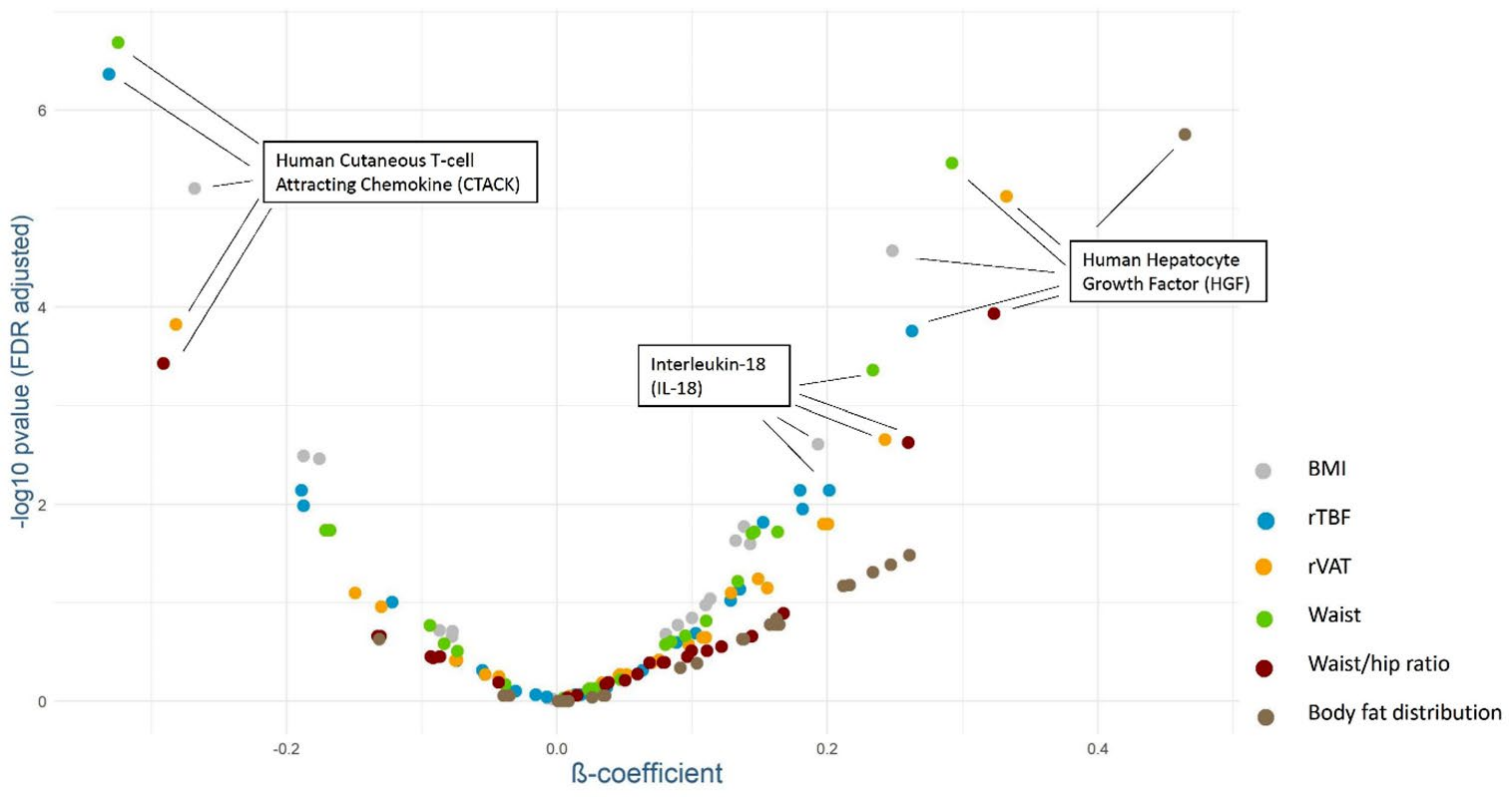
Associations between anthropometric parameters and serum cytokine concentrations. The Linear regression models were adjusted for sex, age, education, smoking status, alcohol consumption, hypothyroidism and corresponding visit (baseline, follow-up). Both, the exposure variables and the outcome were standardized. The figure displays the estimated β-coefficients (X-axis) and the − log_10_ transformed FDR-adjusted p values (Y-axis)

Within both, the mixed models and the linear regression models, the obtained p values were false discovery rate (FDR)-adjusted to control the effect of multiple testing (confidence intervals (CI) displayed in Figs. 3 and 4 and Table S2 were not FDR-adjusted). Results with p values of less than 0.05 were considered to be significant. For all models, the displayed effect estimates (β-coefficient and 95% CI) must be interpreted as the expected change in standardized outcome associated with one standard deviation increase in the exposure variable.

Since loss of follow-up was low and missing observations were very scarce, we performed a complete-case analysis.

All statistical analyses were performed using R version 4.2.1.

## Results

Table 1 displays the baseline characteristics for the total sample and stratified for obesity (BMI ≤ 30 kg/m^2^, BMI > 30 kg/m^2^). Mean age of the cohort was 46.3 years (SD 11.8) and a majority of 68.9% were women. In total, 94 (39.5%) participants had a measured BMI of more than 30 kg/m^2^.

**Table 1:** Baseline characteristics of the total sample and stratified by obesity (BMI ≤ 30 kg/m^2^, BMI > 30 kg/m^2^)

	Total sample (n = 238)	BMI ≤ 30 kg/m (n = 144)	BMI > 30 kg/m^2^ (n = 94)	p value
Age (years)	46.3 (11.8)	45.9 (12)	46.9 (11.6)	0.5301
Sex, female	164 (68.9)	108 (75)	56 (59.6)	0.0178
Body weight (kg)	83.8 (24.4)	67.9 (10.8)	108.1 (18.9)	< 0.001
Body height (cm)	170.8 (9.1)	170.3 (8.5)	171.6 (10)	0.2724
BMI (kg/m^2^)	28.6 (7.6)	23.3 (2.6)	36.6 (5.2)	< 0.001
Relative total body fat (%)	34 (10.5)	28.6 (8.1)	42.6 (7.9)	< 0.001
Relative visceral body fat (%)	2.5 (1.8)	1.5 (1.0)	4 (1.7)	< 0.001
Waist circumference (cm)	93.1 (19.1)	80.5 (9.6)	112.4 (12.8)	< 0.001
Waist-to-hip ratio	0.9 (0.1)	0.8 (0.1)	0.9 (0.1)	< 0.001
Body fat distribution	7.2 (4.8)	5.4 (3.7)	10 (5)	< 0.001
Hypertension	63 (26.5)	21 (14.6)	42 (44.7)	< 0.001
Diabetes mellitus	10 (4.2)	5 (3.5)	5 (5.3)	0.7698
Total alcohol consumption (alcoholic beverages per day)	0.15 (0.05–0.54)	0.15 (0.12–0.54)	0.15 (0.05–0.54)	0.2447
Physical activity				
Medium intensity	0 (0–3)	0 (0–3)	0 (0–2.88)	0.6213
High intensity	0 (0–0)	0 (0–0)	0 (0–0)	0.425
Smoking status				< 0.001
Current smoker	34 (14.3)	13 (9.0)	21 (22.3)	
Never smoker	119 (50)	87 (60.4)	32 (34.0)	
Former smoker	85 (35.7)	44 (30.6)	41 (43.6)	
Vegetarian diet				0.2005
No	214 (89.9)	128 (88.9)	86 (91.5)	
Vegetarian	12 (5)	10 (6.9)	2 (2.1)	
Vegan	12 (5)	6 (4.2)	6 (6.4)	

Mean  (SD) or median (IQR) or n (%)

### Anthropometric parameters and Tregs

Firstly, waist circumference (β-coefficient: − 0.17 [− 0.29, − 0.05], p value: 0.0257) and waist-to-hip ratio (β-coefficient: − 0.20 [− 0.32, − 0.07], p value: 0.0095) showed a significantly inverse associations with the *Treg subset* (as percentage of Tregs in CD4 + T cells) (Fig. 3). rVAT was significantly inversely associated with Tregs only before FDR-adjustment.

Secondly, four parameters were significantly and inversely associated with the *conventional Treg population* (defined as the proportion of CD25 + CD127− cells on all CD45RA− CD4 + T cells): BMI (β-coefficient: − 0.20 [− 0.31, − 0.09], p value: 0.0076), rVAT (β-coefficient: − 0.21 [− 0.34, − 0.08], p value: 0.0196), waist circumference (β-coefficient: − 0.28 [− 0.39, − 0.16], p value: 0.0001) and waist-to-hip ratio (β-coefficient: − 0.27 [− 0.39, − 0.15], p value: 0.0002) (Fig. 3). rTBF and body fat distribution showed significantly inverse associations before, but not after FDR-adjustment.

### Anthropometric parameters and cytokines

Basically, three cytokines were particularly strongly associated with several anthropometric parameters (Fig. 4). On the one hand, the Human cutaneous T-cell Attracting Chemokine (CTACK) was significantly negatively associated with all parameters of body composition apart from body fat distribution. On the other hand, the human hepatocyte growth factor (HGF) showed a significant positive association with all six examined anthropometric parameters. Interleukine-18 (IL-18) was also inversely associated with significant FDR-adjusted p values for all parameters apart from body fat distribution.

In addition to these three cytokines, some more cytokines showed significant association (after FDR-adjustment) with some of the anthropometric parameters: *Human vascular cell adhesion protein 1 [VCAM-1]* was positively associated with BMI, rTBF, and waist circumference; *Human Interleukin-4 [IL-4]* was associated with BMI, rTBF, and waist circumference; *Human Intercellular Adhesion Molecule-1 [Hu ICAM-1]* was related to BMI, rTBF, and waist circumference; *Human Interferon gamma-induced protein [IP-10]* was associated with body fat distribution; *Human Macrophage Inflammatory Protein-1 alpha [MIP-1a]* was associated with BMI, rTBF, rVAT, waist circumference, and body fat distribution; *Human Regulated upon activation, normal T cell expressed and secreted [RANTES]* was related to body fat distribution; *Human Eotaxin [Hu Eotaxin]* was significantly associated with BMI, rTBF, and waist circumference. All results are presented in the supplementary.

## Discussion

In this study, we found significant inverse associations between anthropometric traits and the frequency of Tregs and conventional Tregs. Moreover, three cytokines, CTACK, HGF and IL-18, were identified showing notably strong associations with anthropometric measurements.

### Obesity and Tregs

First, it was noticeable, that for the general Treg population, we found significant association after FDR-adjustment only for the conventional measures waist circumference and waist-to-hip ratio. This comes somehow surprising, since both parameters can be measured easily and without advanced technology. Parameters like rTBF, rVAT or body fat distribution require elaborated measurements (bioelectrical impedance analysis) and therefore are suspected to provide more accurate information about body composition.

Tregs are a specific T cells with immunomodulatory properties, that play a crucial role in mitigation of inflammation and immune response [8]. For a variety of diseases (e.g. autoimmune diseases), Tregs are suspected to be involved in the pathophysiological processes [9]. On the other hand, recent research suggests, that obesity and changes in body composition affect the immune system und in this way are involved in the development of many diseases, but especially metabolic disorders [7, 10–12]. In this study, we show that several parameters of obesity are indeed inversely associated with the relative frequency of Tregs and conventional Tregs.

Few prior studies have investigated the connection between obesity/body composition and Tregs as well, and thereby reported differing results [7]. Some studies reported a (relative) decrease of Tregs in obesity-induced mice [13, 14] and in agreement with this the induction of Tregs improved insulin sensitivity [13]. Furthermore, another study indicated a body-mass dependent depletion of visceral adipose-tissue Tregs in mice and humans [15]. On the other hand, Travers et al. found an increased activation of adipose tissue Tregs in the presence of obesity, but no association for circulating blood Tregs in humans (n = 30) [16]. Similar results were reported by two further studies, showing an increased presence of Tregs in adipose tissue in individuals with obesity [17, 18]. The authors of a review article supposed that these cells accumulate in adipose tissue in order to attenuate obesity-related inflammation [19]. Additionally, van der Weerd et al. examined 13 morbidly obese subjects and 25 lean healthy controls and found that obesity was significantly associated with an increased number of peripheral blood Tregs [20], which is in contrast to the results of the present study.

Moreover, there are also studies indicating, that immunological changes in general and alterations in the Treg biology in particular affect metabolic processes and facilitate obesity. Beppu et al. for instance reported that Tregs promote obesity by Blimp-1-regulated IL-10 secretion, which suppresses adipocyte energy expenditure and thermogenesis [21].

### Obesity and cytokines

In this study, we found a strong positive association between anthropometric measures and IL-18 and HGF. Both results are confirmed by a study from China, which also reported positive associations of IL-18 and HGF with BMI [22]. In the same study, both biomarkers were furthermore associated with an increased risk of cardiovascular diseases [22], which is confirmed by other publications as well [23–25].

IL-18 is a predominantly pro-inflammatory cytokine and supposedly involved in the pathogenesis of a variety of diseases [26]. Likewise, prior studies reported elevated IL-18 levels in individuals with obesity [27–31]. Moreover, it has been found that patients with obesity or type 2 diabetes mellitus are characterized by a IL-18 resistance after stimulation [29]. However, also anti-obesity effects of IL-18 have been described [32]. A recent study by Akimova et al. reported elevated IL-18 levels in overweight or obese lung transplant recipients and suggested an obesity-related Treg impairment induced by IL-18 [28].

HGF is a paracrine cellular growth factor that acts as a pleiotropic cytokine [33]. Next to the study from China mentioned above [22], several other studies have found positive associations between obesity and blood HGF levels as well [34–37]. In a study in mice, however, HGF was found to inhibit diet-induced obesity and to improve insulin resistance [38]. Thus, elevated HGF levels in individuals with obesity might primarily be a compensatory mechanism in reaction to (diet-induced) obesity.

In contrast to IL-18 and HGF, we found a strong inverse association of anthropometric parameters and CTACK, also known as CCL27 [39]. CTACK is a cytokine which is predominantly expressed in the skin and attracts memory T cells [40]. To the best of our knowledge, no prior studies have reported an association between CTACK and obesity so far. Future examinations are necessary to validate our findings.

## Strengths and limitations

This study is characterized by some particular strengths. First, we examined a population-based cohort with a relatively high number of well-phenotyped individuals. For most participants we have one or two follow-up measurements with an overall low drop-out rate. The repeated measurements reduce the susceptibility to short-term immune cell fluctuations caused by infection or illness. Measurements were highly standardized and conducted by qualified and certified staff. The flow cytometry measurements were performed immediately after blood sampling.

Nevertheless, there are some limitations to consider. There is limited quantitative comparability of flow cytometry measurements across different laboratories (e.g. due to a different selection of staining antibodies, different gating strategies or deviating classification of specific subpopulations). In our study, we did not use FoxP3 antibodies for Treg classification, which recently became a commonly used marker for regulatory CD4 + T cells. Another limitation of this study is the missing investigation of Tregs in specific tissues like adipose tissue-resident Tregs. Since the overwhelming majority of our study sample consisted of a typical European population aged 25–65 years, the results might not be generalized to other ethnicities and age-groups. Finally, there might be some unmeasured confounding by disregarding relevant covariables and, as argued above, we cannot make reliable statements about the causality of the observed associations.

## Conclusion

Anthropometric traits and changes in body composition are inversely associated with the relative frequency of Tregs and conventional Tregs. Moreover, the cytokines IL-18 and HGF showed strong positive associations with overweight and obesity. CTACK on the other hand showed a distinct negative association. These findings are essential for a comprehensive understanding of pathophysiological mechanisms underlying obesity and its secondary diseases.

### Supplementary Information

Below is the link to the electronic supplementary material.

Supplementary file1 (DOCX 64 KB)

## Data Availability

The datasets generated during and/or analyzed in the current study are not publicly available due to data protection aspects but are available in an anonymized form from the corresponding author on reasonable request.
