# Supplementary material for: Associations between anthropometric parameters and immune-phenotypical characteristics of circulating Tregs and serum cytokines
Source: Inflamm Res. 2023 Sep 2;72(9):1789–98. doi: 10.1007/s00011-023-01777-1 (PMC10539435; doi:10.1007/s00011-023-01777-1)
Supplement: Supplementary file 1 — Supplementary file1 (DOCX 64 KB) [file 11_2023_1777_MOESM1_ESM.docx]

**Associations between anthropometric parameters and immune-phenotypical characteristics of circulating Tregs and serum cytokines.**

Schmitz T^1^, Freuer D^1^, Linseisen J^1^, Meisinger C^1^

^1^ Epidemiology, Medical Faculty, University of Augsburg, Augsburg, Germany

***Corresponding author:*** Timo Schmitz, Email: [timo.schmitz@med.uni-augsburg.de](mailto:timo.schmitz@med.uni-augsburg.de)

Chair of Epidemiology, University of Augsburg, University Hospital Augsburg

Stenglinstraße 2

86156 Augsburg

Germany

Tel.: +49 821-400-4506

***Table S1****: Definition of Treg subsets by the expression of specific cell surface markers.*

| Cell type | Surface markers |
| --- | --- |
| CD4+ T cells | CD4+ |
| Treg | CD4+ CD25+ CD127- |
| CD4+CD25++ | CD4+ CD25++ |
| CD45RA- | CD4+ CD45RA- |
| CD45RA+ | CD4+ CD45RA+ |
| mTreg | CD4+ CD45RA+ CD25high CD127low |
| nTreg | CD4+ CD45RA- CD25high CD127low |
| CD25++Treg | CD4+ CD25++ CD127- |
| CD25++mTreg | CD4+ CD25++ CD127- CD45RA- |
| CD25++nTreg | CD4+ CD25++ CD127- CD45RA+ |
| CD4+ CD25++ CD127+ CD45RA+ | CD4+ CD25++ CD127+ CD45RA+ |
| CD4+ CD25++ CD127+ CD45RA | CD4+ CD25++ CD127+ CD45RA- |

***Table S2****: Association between cytokines and immunological biomarkers and parameters of obesity using linear regression models*

| ***Cytokine*** | ***NA (%)*** | ***BMI*** | ***rTBF*** | ***rVAT*** | ***Waist circumference*** | ***Waist-to-hip ratio*** | ***body fat distribution*** |
| --- | --- | --- | --- | --- | --- | --- | --- |
| Human vascular endothelial growth factor [Hu VEGF] | 355 (93.42) |  |  |  |  |  |  |
| Human Platelet-derived growth factor BB [Hu PDGF-bb] | 2 (0.53) | -0.01 [-0.11, 0.10], p value: 0.9328 | 0.03 [-0.10,0.15], p value: 0.8090 | 0.10 [-0.03,0.23], p value: 0.2647 | 0.03 [-0.08,0.15], p value: 0.7253 | 0.08 [-0.06,0.22], p value: 0.4017 | 0.16 [-0.01,0.33], p value: 0.1663 |
| Human Interleukin-1 receptor antagonist [Hu IL-1ra] | 248 (65.26) |  |  |  |  |  |  |
| Human Interleukin-2 [Hu IL-2] | 349 (91.84) |  |  |  |  |  |  |
| Human Interleukin-12 p40 [Hu IL-12p40] | 347 (91.32) |  |  |  |  |  |  |
| Human Interleukin-2 receptor antagonist [Hu IL-2Ra] | 126 (33.16) |  |  |  |  |  |  |
| Human Anti-Macrophage migration inhibitory Factor [Hu MIF] | 293 (77.11) |  |  |  |  |  |  |
| Human Vascular cell adhesion protein 1 [Hu VCAM-1] | 1 (0.26) | 0.14 [0.04,0.23],  p value: 0.0168 | 0.15 [0.05,0.26],  p value: 0.0152 | 0.13 [0.01,0.25],  p value: 0.0797 | 0.15 [0.04,0.25],  p value: 0.0190 | 0.07 [-0.06,0.20], p value: 0.4081 | 0.00 [-0.15,0.16], p value: 0.9931 |
| Human Interleukin-4 [Hu IL-4] | 5 (1.32) | -0.18 [-0.28,-0.07], p value: 0.0035 | -0.19 [-0.31,-0.07], p value: 0.0072 | -0.13 [-0.26, 0.00], p value: 0.1093 | -0.17 [-0.28,-0.06],  p value: 0.0183 | -0.13 [-0.27, 0.01],  p value: 0.2185 | 0.03 [-0.14,0.19], p value: 0.9148 |
| Human tumor necrosis factor beta [Hu TNFb] | 1 (0.26) | 0.00 [-0.11,0.10],  p value: 0.9530 | -0.03 [-0.15, 0.09], p value: 0.7875 | 0.03 [-0.10,0.17], p value: 0.6489 | 0.01 [-0.11,0.12], p value: 0.9342 | 0.02 [-0.13,0.16], p value: 0.8734 | 0.14 [-0.04,0.31], p value: 0.2343 |
| Human leukemia inhibitory factor [Hu LIF] | 83 (21.84) | -0.02 [-0.13,0.08], p value: 0.8336 | -0.04 [-0.16,0.09], p value: 0.7841 | -0.02 [-0.15,0.12], p value: 0.8492 | -0.02 [-0.13,0.10], p value: 0.8761 | 0.01 [-0.13,0.15], p value: 0.9185 | 0.06 [-0.11,0.24], p value: 0.7233 |
| Human Interleukin-15 [Hu IL-15] | 376 (98.95) |  |  |  |  |  |  |
| Human Monocyte Chemoattractant Protein-1 [Hu MCP-1(MCAF)] | 2 (0.53) | -0.02 [-0.12,0.09], p value: 0.8534 | -0.01 [-0.13,0.12], p value: 0.9052 | 0.01 [-0.12,0.14], p value: 0.8887 | 0.00 [-0.11,0.12], p value: 0.9342 | 0.12 [-0.02,0.26], p value: 0.2778 | 0.01 [-0.17,0.19], p value: 0.9931 |
| Human tumor necrosis factor alpha [Hu TNF-a] | 58 (15.26) | 0.02 [-0.07,0.12],  p value: 0.8351 | 0.02 [-0.09,0.13],  p value: 0.8625 | 0.05 [-0.07,0.17], p value: 0.5368 | 0.02 [-0.08,0.13], p value: 0.7429 | 0.04 [-0.09,0.17], p value: 0.6461 | 0.16 [0.01,0.32],  p value: 0.1451 |
| Human csCD40 Ligand [sCD40L] | 160 (42.11) |  |  |  |  |  |  |
| Human Cutaneous T-cell Attracting Chemokine [Hu CTACK] | 0 (0.0) | -0.27 [-0.37,-0.17], p value: 0.0000 | -0.33 [-0.44,-0.22], p value: 0.0000 | -0.28 [-0.41,-0.16],  p value: 0.0001 | -0.32 [-0.43,-0.22],  p value: 0.0000 | -0.29 [-0.43,-0.16],  p value: 0.0004 | -0.13 [-0.30,0.04], p value: 0.2343 |
| Human Interleukin-1 alpha [Hu IL-1a] | 249 (65.53) |  |  |  |  |  |  |
| Human Interleukin-18 [Hu IL-18] | 8 (2.11) | 0.19 [0.09,0.30],  p value: 0.0024 | 0.20 [0.08,0.32],  p value: 0.0072 | 0.24 [0.11,0.37],  p value: 0.0022 | 0.23 [0.12,0.35],  p value: 0.0004 | 0.26 [0.12,0.40],  p value: 0.0023 | 0.21 [0.04,0.39],  p value: 0.0674 |
| Human stromal cell-derived factor-1 [Hu SDF-1a] | 1 (0.26) | -0.09 [-0.19,0.02], p value: 0.1915 | -0.12 [-0.24,0.00], p value: 0.0990 | -0.08 [-0.21,0.05], p value: 0.3851 | -0.08 [-0.20,0.03], p value: 0.2613 | -0.13 [-0.27,0.01], p value: 0.2185 | -0.04 [-0.21,0.13], p value: 0.8798 |
| Human Intercellular Adhesion Molecule-1 [Hu ICAM-1] | 1 (0.26) | 0.13 [0.04,0.23],  p value: 0.0234 | 0.18 [0.07,0.29],  p value: 0.0072 | 0.15 [0.03,0.27],  p value: 0.0571 | 0.14 [0.04,0.25],  p value: 0.0199 | 0.10 [-0.03,0.23], p value: 0.3067 | 0.04 [-0.12,0.19], p value: 0.8798 |
| Human Granulocyte macrophage-colony stimulating factor [Hu GM-CSF] | 323 (85.0) |  |  |  |  |  |  |
| Human Stem cell factor [Hu SCF] | 1 (0.26) | -0.04 [-0.15,0.06], p value: 0.5838 | -0.05 [-0.17,0.07], p value: 0.5353 | -0.04 [-0.17,0.09], p value: 0.5634 | -0.04 [-0.15,0.08], p value: 0.6787 | 0.04 [-0.11,0.18], p value: 0.6751 | 0.00 [-0.17,0.18], p value: 0.9931 |
| Human Interleukin-8 [Hu IL-8] | 310 (81.58) |  |  |  |  |  |  |
| Human Macrophage Inflammatory Protein-1 beta [Hu MIP-1b] | 1 (0.26) | 0.03 [-0.07,0.14],  p value: 0.6984 | 0.01 [-0.11,0.14],  p value: 0.8643 | 0.11 [-0.03,0.24], p value: 0.2245 | 0.05 [-0.07,0.16], p value: 0.6034 | 0.05 [-0.09,0.19], p value: 0.6123 | 0.22 [0.04,0.39],  p value: 0.0664 |
| Human Interleukin-10 [Hu IL-10] | 335 (88.16) |  |  |  |  |  |  |
| Human Monocyte Chemoattractant Protein-3 [Hu MCP-3] | 377 (99.21) |  |  |  |  |  |  |
| Human Interferon gamma [Hu IFN-g] | 55 (14.47) | 0.10 [-0.01,0.21],  p value: 0.1429 | 0.10 [-0.02,0.23],  p value: 0.2046 | 0.11 [-0.02,0.24], p value: 0.2245 | 0.09 [-0.02,0.21], p value: 0.2160 | -0.04 [-0.19,0.10], p value: 0.6461 | 0.14 [-0.04,0.31], p value: 0.2343 |
| Human Stem Cell Growth Factor beta [Hu SCGFb] | 1 (0.26) | 0.02 [-0.09,0.12],  p value: 0.8534 | 0.04 [-0.08,0.16],  p value: 0.7208 | 0.05 [-0.08,0.17], p value: 0.5520 | 0.03 [-0.09,0.14], p value: 0.7429 | 0.08 [-0.06,0.22], p value: 0.4017 | 0.16 [0.00,0.33],  p value: 0.1663 |
| Human Interleukin-16 [Hu IL-16] | 1 (0.26) | 0.08 [-0.02,0.19],  p value: 0.2092 | 0.06 [-0.06,0.18],  p value: 0.4833 | 0.07 [-0.06,0.20], p value: 0.4092 | 0.08 [-0.03,0.19], p value: 0.2658 | 0.11 [-0.03,0.25], p value: 0.3067 | 0.10 [-0.07,0.28], p value: 0.4124 |
| Human fibroblast growth factor basic [Hu FGF basic] | 131 (34.47) |  |  |  |  |  |  |
| Human Interleukin-13 [Hu IL-13] | 174 (45.79) |  |  |  |  |  |  |
| Human Interleukin-1 beta [Hu IL-1b] | 71 (18.68) | -0.02 [-0.13,0.08], p value: 0.8336 | -0.02 [-0.13,0.10], p value: 0.8614 | 0.04 [-0.09,0.16], p value: 0.6661 | -0.01 [-0.13,0.10], p value: 0.8874 | 0.01 [-0.13,0.15], p value: 0.9185 | 0.13 [-0.04,0.29], p value: 0.2651 |
| Human growth-regulated oncogene-alpha Protein [Hu GROa] | 302 (79.47) |  |  |  |  |  |  |
| Human Interleukin-6 [Hu IL-6] | 253 (66.58) |  |  |  |  |  |  |
| Human Interferon gamma-induced protein [Hu IP-10] | 1 (0.26) | 0.11 [0.01,0.22],  p value: 0.0914 | 0.14 [0.02,0.26],  p value: 0.0732 | 0.20 [0.07,0.33],  p value: 0.0159 | 0.13 [0.02,0.25],  p value: 0.0607 | 0.10 [-0.04,0.24], p value: 0.3537 | 0.26 [0.09,0.43],  p value: 0.0328 |
| Human Granulocyte Colony Stimulating Factor [Hu G-CSF] | 35 (9.21) | -0.08 [-0.18,0.03], p value: 0.2200 | -0.07 [-0.19,0.05], p value: 0.3884 | -0.05 [-0.18,0.08], p value: 0.5368 | -0.07 [-0.19,0.04], p value: 0.3103 | -0.09 [-0.23,0.05], p value: 0.3537 | 0.03 [-0.14,0.21], p value: 0.8798 |
| Human Hepatocyte Growth Factor [Hu HGF] | 1 (0.26) | 0.25 [0.15,0.35],  p value: 0.0000 | 0.26 [0.15,0.38],  p value: 0.0002 | 0.33 [0.21,0.46],  p value: 0.0000 | 0.29 [0.18,0.40],  p value: 0.0000 | 0.32 [0.19,0.46],  p value: 0.0001 | 0.46 [0.30,0.63],  p value: 0.0000 |
| Human Nerve growth factor beta [Hu b-NGF] | 346 (91.05) |  |  |  |  |  |  |
| Human Interleukin-3 [Hu IL-3] | 362 (95.26) |  |  |  |  |  |  |
| Human Interleukin-5 [Hu IL-5] | 298 (78.42) |  |  |  |  |  |  |
| Human TNF-Related Apoptosis Inducing Ligand [Hu TRAIL] | 134 (35.26) |  |  |  |  |  |  |
| Human Macrophage Inflammatory Protein-1 alpha [Hu MIP-1a] | 31 (8.16) | 0.14 [0.04,0.25],  p value: 0.0252 | 0.18 [0.06,0.30],  p value: 0.0111 | 0.20 [0.07,0.33],  p value: 0.0159 | 0.16 [0.05,0.28],  p value: 0.0190 | 0.17 [0.03,0.31],  p value: 0.1278 | 0.25 [0.07,0.42],  p value: 0.0409 |
| Human Regulated upon activation, normal T cell expressed and secreted [Hu RANTES] | 1 (0.26) | 0.11 [0.00,0.22],  p value: 0.1053 | 0.13 [0.01,0.25],  p value: 0.0946 | 0.16 [0.02,0.29],  p value: 0.0709 | 0.11 [-0.01,0.23], p value: 0.1526 | 0.14 [0.00,0.29],  p value: 0.2185 | 0.23 [0.06,0.41],  p value: 0.0489 |
| Interleukin-31 [IL-31] | 373 (98.16) |  |  |  |  |  |  |
| Human Interleukin-7 [Hu IL-7] | 364 (95.79) |  |  |  |  |  |  |
| Interferon Alpha 2 [Hu IFN-a2] | 375 (98.68) |  |  |  |  |  |  |
| Human Eotaxin [Hu Eotaxin] | 1 ( 0.26 ) | -0.19 [-0.29,-0.08], p value: 0.0032 | -0.19 [-0.31,-0.07], p value: 0.0103 | -0.15 [-0.28,-0.02],  p value: 0.0797 | -0.17 [-0.29,-0.06],  p value: 0.0183 | -0.09 [-0.24,0.05], p value: 0.3619 | -0.04 [-0.21,0.14], p value: 0.8798 |
| Human Macrophage Colony Stimulating Factor 1 [Hu MCSF / Hu M-CSF] | 5 (1.32) | 0.09 [-0.01,0.19],  p value: 0.1682 | 0.09 [-0.03,0.21],  p value: 0.2537 | 0.08 [-0.05,0.20], p value: 0.3813 | 0.08 [-0.03,0.20], p value: 0.2483 | 0.06 [-0.08,0.20], p value: 0.5296 | 0.09 [-0.08,0.26], p value: 0.4559 |
| Human Interleukin-17 [Hu IL-17] | 228 (60.0) |  |  |  |  |  |  |
| Human Interleukin-12 p70 [Hu IL-12(p70)] | 367 (96.58) |  |  |  |  |  |  |
| Human Interleukin-9 [Hu IL-9] | 1 (0.26) | 0.02 [-0.09,0.12],  p value: 0.8534 | -0.02 [-0.14,0.11], p value: 0.8643 | 0.05 [-0.08,0.19], p value: 0.5368 | 0.02 [-0.09,0.14], p value: 0.7583 | 0.01 [-0.14,0.15], p value: 0.9185 | 0.16 [-0.01,0.34], p value: 0.1663 |
| Human Monokine induced by gamma interferon [Hu MIG] | 0 (0.0) | -0.08 [-0.17,0.02], p value: 0.1950 | -0.06 [-0.16,0.05], p value: 0.4833 | -0.07 [-0.19,0.05], p value: 0.3813 | -0.09 [-0.20,0.01], p value: 0.1699 | -0.09 [-0.21,0.04], p value: 0.3537 | 0.00 [-0.16,0.16], p value: 0.9931 |
